# Chemical Structure-Biological Activity Models for Pharmacophores’ 3D-Interactions

**DOI:** 10.3390/ijms17071087

**Published:** 2016-07-08

**Authors:** Mihai V. Putz, Corina Duda-Seiman, Daniel Duda-Seiman, Ana-Maria Putz, Iulia Alexandrescu, Maria Mernea, Speranta Avram

**Affiliations:** 1 Laboratory of Computational and Structural Physical-Chemistry for Nanosciences and QSAR, Department of Biology-Chemistry, West University of Timişoara, Pestalozzi Str. 16, RO-300115 Timisoara, Romania; cori_mam@yahoo.com (C.D.-S.); putzanamaria@yahoo.com (A.-M.P.); 2 Laboratory of Renewable Energies-Photovoltaics, R & D National Institute for Electrochemistry and Condensed Matter, Dr. A. Paunescu Podeanu Str. No. 144, RO-300569 Timisoara, Romania; 3 Department of Medical Ambulatory, and Medical Emergencies, University of Medicine and Pharmacy “Victor Babes”, Avenue C. D. Loga No. 49, RO-300020 Timisoara, Romania; duda.seiman@cardiologie.ro; 4 Institute of Chemistry Timișoara of the Romanian Academy, 24 Mihai Viteazul Bld., RO-300223 Timisoara, Romania; 5 Department of Anatomy, Animal Physiology and Biophysics, Faculty of Biology, University of Bucharest, Str. 91-95th Independentei, RO-050095 Bucharest, Romania; iuliaaim@yahoo.com (I.A.); maria.mernea@bio.unibuc.ro (M.M.); speranta.avram@gmail.com (S.A.)

**Keywords:** quantitative structure-activity relationship (QSAR), multi-linear correlation, statistical correlation, chemical stericity, ligand binding, cross-validation, hydrophobicity, van der Waals interaction, molecular mechanism, drug design

## Abstract

Within medicinal chemistry nowadays, the so-called pharmaco-dynamics seeks for qualitative (for understanding) and quantitative (for predicting) mechanisms/models by which given chemical structure or series of congeners actively act on biological sites either by focused interaction/therapy or by diffuse/hazardous influence. To this aim, the present review exposes three of the fertile directions in approaching the biological activity by chemical structural causes: the special computing trace of the algebraic structure-activity relationship (SPECTRAL-SAR) offering the full analytical counterpart for multi-variate computational regression, the minimal topological difference (MTD) as the revived precursor for comparative molecular field analyses (CoMFA) and comparative molecular similarity indices analysis (CoMSIA); all of these methods and algorithms were presented, discussed and exemplified on relevant chemical medicinal systems as proton pump inhibitors belonging to the 4-indolyl,2-guanidinothiazole class of derivatives blocking the acid secretion from parietal cells in the stomach, the 1-[(2-hydroxyethoxy)-methyl]-6-(phenylthio)thymine congeners’ (HEPT ligands) antiviral activity against Human Immunodeficiency Virus of first type (HIV-1) and new pharmacophores in treating severe genetic disorders (like depression and psychosis), respectively, all involving 3D pharmacophore interactions.

## 1. Introduction

The quantitative structure-activity relationship (QSAR) method has wide applications in medical chemistry, one of them being the design of new drugs. The study of the interactions between macromolecules (proteins, membrane receptors) and their ligand drugs is very important for many scientific fields, like psychiatry, chemistry, pharmacology or biochemistry. Because the molecular mechanisms of many severe genetic disorders are unclear, it is necessary to apply fast methods (e.g., in silico) in order to analyze and predict the biological activity of new pharmacophores, able to reduce the genetic disorders’ symptoms. For this, new techniques, such as computational chemistry [1,2], high-throughput screening [3] and combinatorial chemistry [4], are available. Basically, the theoretical aspects of these techniques include the consideration that a potent ligand fits in the active site of its target protein or membrane receptor, while an inactive ligand does not. Many in silico methods that are useful for investigating ligand–protein interactions require the knowledge of proteins’ or receptors’ crystallographic structures [5]. Unfortunately, such structures are not available for all potential drug targets. Usually, when the pharmacological effects of psychiatric drugs (e.g., neuroleptics, antidepressants) are discussed, the dopamine, adrenergic and serotonin receptors are taken into account [6,7,8]. The 3D structures of these membrane receptors are not available. In such conditions, when the structures of the receptors or proteins are unknown, QSAR methods remain an appropriate option to predict the pharmacological effects of psychiatric drugs. Thus, QSAR methods are required in order to elucidate the possible mechanisms underlying genetic disorders like psychiatric or others disorders and to design new pharmacophores useful in their treatment.

All QSAR methods consider that drugs’ macroscopic properties (e.g., biological activity) are induced by their molecular structure, and every change in the molecular structure leads to a change of these properties [9,10]. A QSAR model generally takes the form of a linear equation, under conceptual form:

Biological activity (log1/*K*_i_) = const + (*c*_1_ × *p*_1_) + (*c*_2_ × *p*_2_) + (*c*_3_ × *p*_3_) + ... (1a)

where the parameters *p*_1_ through *p*_n_ may be molecular volume, dipole moment, etc., while being computed for each molecule in the series; the coefficients *c*_1_ through *c*_n_ are currently calculated by fitting variations in the parameters against the biological activity.

The good correlation between observed and predicted biological activities allows the design of new derivatives starting from a template compound (the most active molecule of the study set) with improved pharmacokinetic properties. QSAR performed before drug synthesis allows the identification of the most potent compounds worth synthesizing, which significantly decreases the time and cost of the procedures [11]. Generally, 2D and 3D-QSAR methods consider the structural changes that influence the biological activity as being dependent on three important, potentially independent, properties, namely: electronic, steric and hydrophobic [10,12]. Advanced 3D-QSAR methods aim at identifying spatial features shown by the similarity or differences between electrostatic and steric force fields of the active site vs. their ligands, at the 3D level of macromolecule structural representation [13,14]. The selection of biological data, their range and distribution are critical to the successful development of a QSAR model. It is preferred to take into account a range of biological activities as wide as possible (a minimum of three log units) [15].

In this context, the actual review is organized as follows: it opens with a new paradigm of modeling the structure-activity by the so-called spectral or algebraic approach (special computing trace of the algebraic structure-activity relationship (SPECTRAL-SAR)) [16,17]; this is followed by a conceptual-computational basis of modeling the 3D interaction by the aid of the minimal topological/sterical difference (MTD) [18,19] that serves also as a precursor of the modern 3D methods of comparative molecular field analyses (CoMFA) and comparative molecular similarity indices analysis (CoMSIA), able to approach the complex problems of assessing genetic diseases on a specific molecular interaction [20,21]. Conclusions summarize the actual status of modeling the pharmacophores’ 3D interaction in the view of solving the bio-chemical interaction in a universal structural manner.

## 2. The Special Computing Trace of Algebraic Structure-Activity Relationship (SAR) Method

### 2.1. General Special Computing Trace of the Algebraic Structure-Activity Relationship (SPECTRAL-SAR) Algorithm

Quantitative structure-activity relationships (QSARs) have proven to be a successful attempt to describe chemical-biological interactions because of their capacity to provide the analytical predictive equation applied to analogues of certain classes of compounds, within an application domain linking the chemical causes with their bio-, eco- and pharmaco-effects [22,23,24,25,26,27,28,29,30,31,32,33,34,35,36,37]. It appears to be *a* *macroscopic* equation, but this observation is countered, since it falls out of commonly-accepted forms for natural phenomena according to which an effect under study is modeled by an exponential, polynomial, logarithmic or trigonometric function. Conversely, the custom linearity and multi-linearity dependence of a QSAR study using the recorded activity by the molecular M-descriptors as the following equation:

(1b) $$A=a_{0}+a_{1}X_{1}+a_{2}X_{2}+...+a_{M}X_{M}$$

finely respects the *superposition principle* in quantum matter, modeling at the *micro-universe* level the substance’ structure and evolution; consequently, it is right and correct to point out that as far as the states are each represented by independent (orthogonal) descriptors/properties, their superposition into a molecular architecture appropriately produces, by an optimum algorithm, the projection coefficients ($a_{0},a_{1},...,a_{M}$) driving the observed state for (measured) activity, therefore explained by the so-called Banach chemical-property space [16,38,39,40,41,42]:

(2) $$|A\rangle =a_{0}|X_{0}\rangle +a_{1}|X_{1}\rangle +a_{2}|X_{2}\rangle +...+a_{M}|X_{M}\rangle$$

with $|X_{0}\rangle ={\hat{1}}_{N}=|11...1_{N}\rangle$ being the unitary vector with *N*-dimensions as corresponding to the *N*-compounds in the congener pool of toxicants.

This idea was entirely employed and exploited by the already consecrated SPECTRAL-SAR vectorial version of QSAR, where biological activity is reloaded as a vectorial decomposition into an orthogonal multi-dimensional space of molecular descriptors. Beyond the algebraic consequences in dealing with correlation in absolute manner, i.e., by reforming the statistical least search of errors, the “quantum” or Banach vectorial approach of QSAR yields two major ideas with considerable closeness to the ultimate goal of any QSAR study: establishing the mechanistic scenario driving the chemical-biological interaction under concern [17,43,44,45,46]:

- The orthogonality constraint of the molecular descriptors’ states involved in Equation (2),
  (3) $$|X_{1}\rangle ⊥|X_{i}\rangle ⊥|X_{j}\rangle ,\;\forall i,j=2,...,M$$
  also restrains the classes of molecular descriptors to be considered; for instance, the electronegativity and chemical hardness are ideal candidates because of their orthogonal and complementary conceptually nature [47,48];
- Introducing the variational principle selecting from the pool of various predicted *endpoints* of Equation (2), from those of linear, bilinear, up to those that are multi-linear in nature, e.g.,
  (4a) $$|Y_{Ii}\rangle =y_{I0}|X_{0}\rangle +y_{1i}|X_{1i}\rangle ,\;i=1,...,M$$
  (4b) $$|Y_{IIi}\rangle =y_{II0}|X_{0}\rangle +y_{II1i}|X_{1i}\rangle +y_{II2i}|X_{2i}\rangle ,\;i=1,...,M$$
  …
  (4c) $$|Y_{M}\rangle =y_{M0}|X_{0}\rangle +y_{M1}|X_{1}\rangle +y_{M2}|X_{2}\rangle +...+y_{MM}|X_{M}\rangle$$
  with the general form given by the SPECTRAL-SAR algorithm [16,17,42]:
  (4d) $$\left|\begin{array}{ccccccc} |Y\rangle & \omega_{0} & \omega_{1} & \cdots & \omega_{k} & \cdots & \omega_{M}\\ |X_{0}\rangle & 1 & 0 & \cdots & 0 & \cdots & 0\\ |X_{1}\rangle & r_{0}^{1} & 1 & \cdots & 0 & \cdots & 0\\ \vdots & \vdots & \vdots & \vdots & \vdots \\ |X_{k}\rangle & r_{0}^{k} & r_{1}^{k} & \cdots & 1 & \cdots & 0\\ \vdots & \vdots & \vdots & \vdots & \vdots \\ |X_{M}\rangle & r_{0}^{M} & r_{1}^{M} & \cdots & r_{k}^{M} & \cdots & 1 \end{array}\right|=0$$
  (4e) $$\omega_{k}=\frac{\langle \Omega_{k}|Y\rangle }{\langle \Omega_{k}|\Omega_{k}\rangle },\;k=\bar{0,M}$$
  (4f) $$r_{i}^{k}=\frac{\langle X_{k}|\Omega_{i}\rangle }{\langle \Omega_{i}|\Omega_{i}\rangle }$$
  with the vectors $|\Omega_{0}\rangle$, $|\Omega_{1}\rangle$, …, $|\Omega_{k-1}\rangle$ orthogonally constructed, i.e.,
  (4g) $$\langle \Omega_{0}|\Omega_{k}\rangle =0$$
  assuring that $|\Omega_{0}\rangle$ and $|\Omega_{k}\rangle$ are orthogonal, along the general Gram–Schmidt expressions:
  (4h) $$|\Omega_{k}\rangle =|X_{k}\rangle -\sum_{i=0}^{k-1}r_{i}^{k}|\Omega_{i}\rangle$$
  so that the vector $|\Omega_{k}\rangle$ is orthogonal on all previous ones.

Finally, the shortest path is found throughout the variational procedure imposed among all possible connected paths, namely:

(5) $$0=\delta {\left\{|Y_{Ii}\rangle ,|Y_{IIi}\rangle ,...,|Y_{M}\rangle \right\}}_{i=\bar{1,M}}$$

It also releases with the identification of the above searched mechanism explaining the interaction by the appropriate identification of the molecular descriptors appearing in the least path of Equation (5) [17,43,44,45,46].

### 2.2. Results of SPECTRAL-SAR in Pharmacophores’ 3D-Interaction

It is important to underline that the SPECTRAL-SAR procedure originally embraces the desiderate and principle No. 5 for any QSAR study of the Organization for Economic Cooperation and Development (OECD) [49,50], namely fully addressing the mechanistic picture of the molecular impact/interaction on the biological/organismal site. It was, however, innovatively combined with chemical reactivity principles of electronegativity and hardness, as well as with the logistic framework of ligand-receptor interaction (in its turn, generalizing the Michaelis–Menten enzyme kinetics) [51].

To this end, whenever on the molecular structure of the ligand L or on enzyme more X substituents are coupled, their effects can be regarded in an additive quantum (superposition) way or, on the contrary (classically), each of the substituents is treated separately. Because it may be difficult to characterize quantitatively a certain structure, it is obvious why there is a large amount of parametric scales. Paradigmatically, for the transportation parameter, the logarithm of the partition coefficient of the bioactive compound (L) is used, and almost in all cases, it is determined as a water to 1-octanol ratio simulation of the lipidic hydrophobic membrane, among other derived parameters. On the other hand, the electronic structure of bioactive compounds is customarily quantified as starting from the thermodynamic model using the substituent constants as the well-known σ-Hammett benchmark [52], supporting also further refining [53]. These are nevertheless, in most cases, the adequate parameterization possibilities for including the electronic effects’ parameterization; for their use, there are large computer databases delivering such constants when numerous substituents are considered [54]. For a further structural insight, one needs an alternative approach employing quantum reactivity indices, like electronegativity, chemical hardness, bonding orders, charges on an atom series, etc.

**Figure 1:**
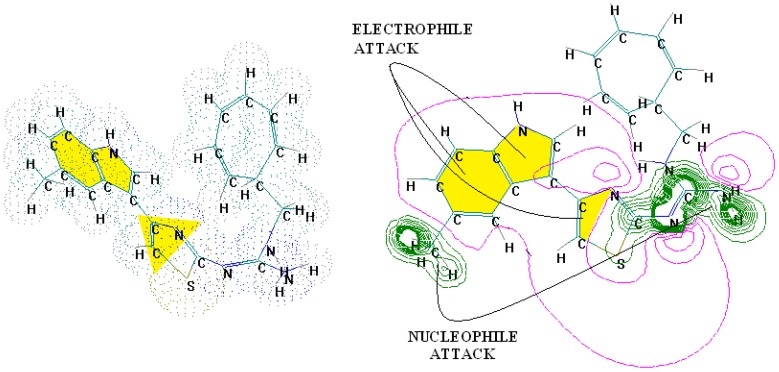
The electronic basins (**left**) and the associated electrostatic potential contours (**right**) for 4-(5-methylindolyl)-2-methylheptylguanidinothiazole, displaying optimized maximum electrophilic activity by the predicted special computing trace of the algebraic structure-activity relationship (SPECTRAL-SAR) mechanism action [56]. Green lines correspond with highest occupied molecular orbitals (HOMO), magenta with lowest unoccupied molecular orbitals (LUMO) with the yellow shapes marked as key fragments for chemical frontier reactivity.

The SPECTRAL-SAR algorithm was tested more explicitly in relation to the 3D structural information through employing the least spectral paths (say α, β and γ spectral paths, for the three parameters’ correlation in biological activity) connecting the considered endpoint computed models for a paradigmatic correlation analysis, upon Equation (5), and applied on a selected series of 4-indolyl, 2-guanidinothiazole derivatives with a potential role in gastric anti-secretor activity, by inhibiting the gastric H^+^/K^+^ ATPase proton pump in parietal cells of the stomach [55]. Note that in molecular design, we are forecasting molecules that optimally interact through controlled or predicted mechanisms, constructed on interpretable molecular indices, e.g., as assumed by the Hansch correlation scheme (i.e., seen as a combination of hydrophobicity Log*P* controlling the cellular transduction, the polarizability (POL) controlling the electrostatic interaction, being overall tuned by total energy, Etot, so accounting for the degree of covalent bindings). The performed study in this area obtained the series of maximum and minimum residue values across α, β and γ spectral paths accordingly ordered from the smallest ahead, while identifying the observed activity for each molecule in those series, respectively; the molecular null and negative observed activities have an unfavorable (or detrimental) contribution to binding. The search continued until the molecule 4-(5-methylindolyl)-2-methylheptylguanidinothiazole (Figure 1) was found with the closest to the higher observed activity [56].

Note that in the case that two candidate molecules are found with the same observed activity the preferred one will be that belonging to the spectral pathway displaying a superior degree (α > β > γ); if they further belong to the same path (i.e., they both feature equal positive and negative residual values against the same observed activity), then the common structure resulting from their superposition will be considered as the *designed* active molecular fragment or site by the spectral path analysis. This kind of completing of the S-SAR algorithm predicts the 3D molecular structure performing the optimum attack on the envisaged biological sites by certain (controlled) structural mechanism models. It may be appropriately called the 3D-SSAR method. In the reported case of Figure 1, the described 3D-SPECTRAL-SAR method shows a large area of *electrophilic interaction* (so governed by polarization), which eventually complements by inhibiting the *protonation* role of H^+^K^+^-ATPase, as one should optimally predict [57,58,59,60].

Even further, when the non-linear correlation (interactions) are involved in more complex (open) biological systems, Thom’s theory of catastrophes appears as one of the most preeminent mathematical theories modeling open-system dynamics. It is widely used because of its simple yet valuable modeling of the system-environment interaction; moreover, it is inclusive towards phenomena, such as equilibrium/steady state and/or life cycles [61]. Biological systems are the first submitted to catastrophe modeling because of their action-reaction causal response to various natural stimuli and feedback to the imposed constraining limits. The reactions of organisms against vital toxicological threats were accordingly developed, e.g., into the survival attractor concept as driven by the butterfly bifurcation phenomenology, also closely related to the cusp catastrophe [62]. In its turn, the cusp catastrophe may be further related to the physiological processes of predation and generation, thus giving theoretical biology support to Heidegger’s philosophical concept of *entity*; it may be thus regarded as a tool in translating the ontological entities into computer language [63]. In this context, Jungian psychology may be proclaimed as entering the topological phase approach by modeling personal unconscious and conscious minds by the swallowtail catastrophe [64]. As a consequence, neuro-self-organization is correspondingly advanced by the reduction of the archetypal precursor of epileptic seizures to cusp synergetics [65]. Lastly, but not least, the catastrophe approach enters chemistry through the need to unitarily characterize chemical bonding through elementary processes, leading to the so-called bonding evolution theory; it also triggered the reformulation of the electronic localization functions [66,67]. Catastrophe theory was nevertheless successfully grounded on Hilbert space modeling with the aid of density matrix and non-linear evolution-specific tools including non-commutative (quantum open) systems [68].

## 3. The Minimal Topological Difference Method

### 3.1. General MTD Algorithm

The concept of “key-into-lock” promoted by Fischer is a very well-known, and it responds in an essential way to the MTD requirements. Thus, the philosophy of MTD techniques is based on the fact that a ligand *L*_i_ has an ideal correspondent at the level of the receptor (binding site). Translated into the MTD language, this principle would acknowledge that in the case of a degree of misfit, the larger this degree of misfit is, the lower is the biological activity *A*_i_ of that ligand. The MTD technique was developed by the QSAR group Timisoara [69,70].

In simple terms, the MTD method can be perceived by means of volumes. Thus, when the affinity of the ligand at the binding site of the receptor is low, the receptor registers an augmentation of its un-overlapped volume, in a commensurate manner.

In order to achieve the MTD steric parameters, the molecular stereochemistry is compared using two types of interventions:

- molecular superposition, which identifies similarities within a series of molecules or describes a “pharmacophoric constellation” of atoms,
- and identifying and describing the positions that are equivalent.

A “hyperstructure” is obtained by combining these equivalent positions and the bonds between them. The “hyper-molecule” includes both topological and geometrical aspects.

In a series of *N* molecules *L*_i_ (*i* = 1, 2,…, *N*) in which their activities *A*_i_ are known, in order to identify the most biologically-active molecule, one should apply specific molecular modeling techniques to provide the structure-activity correlation equation. With respect to the MTD philosophy, this correlation equation measures quantitatively the level of mismatch between the ligand and the cavity of the receptor, materialized by the steric/topologic differences MTD_i_ [71,72]. As stated before, the MTD technique requires firstly to build, and, afterwards to optimize the so-called hyper-molecule (**H**). The “contracted” hydrogen atoms are considered to build the hyper-molecule (**H**): in this phase, second or higher period atoms X or XH_n_ are considered for superposition. The superposition stage requires that molecules with a fixed orientation have to be taken into account, so that the superposition process takes place in an atom-over-atom manner. An *S* active and rigid molecule is taken as the starting molecule, over which all other molecules will be maximally superimposed, which means that the distances less than 0.5 Å after maximum superposition are comprised in the same vertex *j* in the **H** hyper-molecule. Completing the superposition process, a number of *j* vertices results$j=\bar{\text{1,M}}$. The *j* vertices express within the R*L_i_ complex the estimation of the atoms’ position. The edges in this complex are defined through the valence bonds between atoms. The interaction between ligands and receptors provides an activated R*L_i_ complex, like:

*R*(aq) + *L*_i_(aq) → R*L_i_(aq) + nH_2_O (6)

where R* represents the active conformation of the receptor, while a *L*_i_ ligand binds to that receptor.

To simplify the description of the obtained **H** hyper-molecule, hydrogen atoms are not represented. Therefore, each *L*_i_ ligand implies a “vectorial” identification of the {*x*_ij_} type, where *x*_ij_ = 0, 1. We have *x*_ij_ = 0 when the *j* vertex in *L*_i_ identifies no occupancy. In opposition, *x*_ij_ = 1. The minimal topological difference MTD_i_ of molecule “*i*” has the following shape [18,19,69,70,71,72]:

(7) $${\text{MTD}}_{i}=s+\sum_{j=1}^{M}\varepsilon_{j}\cdot x_{\text{ij}};\;i\;=\;1,\;\ldots ,\;N$$

where ε_j_ concerns the situations of vertices in relation to the receptor cavity. ε_j_ = −1 is the benefic situation for a sustained biological activity (vertices are attributed to the receptor cavity); ε_j_ = 0 expresses the situation when vertices are situated externally from the receptor cavity (irrelevant); ε_j_ = +1 describes the situation when vertices are attributed to the receptor walls, expressing a detrimental biological activity. The s parameter in Equation (7) takes into account all vertices in the receptor cavity (ε_j_ = −1). $\sum_{j=1}^{M}\varepsilon_{j}\cdot x_{\text{ij}}$ in Equation (7) is the sum of the situations of occupancy and non-occupancy. Equation (7) represents the quantitative representation of the “steric relation between each ligand “*i*” of the total tested *N* respecting the available *M* vertices of receptor *R* in the course of bonding”. The mismatch in terms of steric aspects within the *L*_i_ molecule is provided by the count of the vertices with no cavity occupancy in **H** and the count of the vertices with wall occupancy. Assuming that there are more conformations with low energy, that conformation will be used in a version of Equation (7) that shows the best match to the receptor site (*k* = number of conformations of low energy, where *k* = 1,…,*C*_i_ and *C*_i_ is the number of conformations of one *I* molecule) [18,19,69,70,71,72]:

(8) $${\text{MTD}}_{i}=\underset{k}{\text{min}}\left(s+\sum_{j=1}^{M}\varepsilon_{j}\cdot x_{\text{ij}}^{\text{[k]}}\right)$$

Thus, the MTD technique provides a regression equation of the following form:

(9) $$A=\alpha_{0}+\alpha_{1}\cdot P_{1}+\alpha_{2}\cdot P_{2}+\cdot \cdot \cdot -\beta \cdot \text{MTD}$$

where *P*_i_, *i* = 1, 2, …, define additional or alternative structural parameters that can be considered within the optimization stages or after the S* receptor chart has been defined according to the optimized MTD* values.

Actual data provide the idea that the steric mismatch (misfit) can be adjusted with an energy that varies proportionally to the corresponding volume mismatch. In Equation (9), the minus sign points out that there is a direct relation between biological activity and the amount of ligand steric misfit, while the first decreases in relation to the increase of the latter.

To start an optimization process of a given series of molecules, it is necessary to define an initial $\left\{\varepsilon_{j}^{0}\right\}$ attribution set corresponding to the beginning chart *S*^0^. The next step is to obtain the ${\text{MTD}}_{i}^{0}$ values, the regression coefficients in Equation (9) (multi-linear regression) and the associated correlation coefficient $r^{0}$. The next step consists of the one by one modification of *ε*_j_ attributions. A number of 2M mono-substituted charts will be obtained, combining the MTD_i_ values with the 2M correlation equations of type Equation (9) and their correlation coefficients. The next step considers the mono-substituted chart with the highest correlation coefficient *r*. This algorithm is repeated until it is observed that the regression coefficient *r* shows no more increase tendency. At this time, the final $\left\{\varepsilon_{j}\right\}$ set of values is obtained, $j=\bar{\text{1,M}}$, obtaining thus the optimized receptor chart, S*. This optimized receptor chart is used in Equation (7), providing the optimized predictor variables MTD* for the QSAR problem [18].

### 3.2. Results of MTD in Pharmacophores’ 3D-Interaction

**Figure 2:**
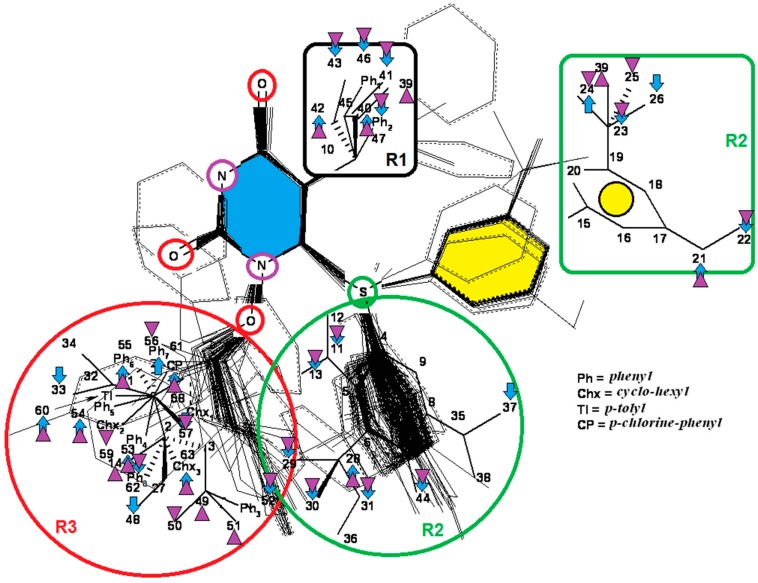
1-[(2-hydroxyethoxy)-methyl]-6-(phenylthio)thymine (HEPT) structure with superimposition of 79 derivatives with minimum energy conformations; these are (marked in blue, in the center) the possible positions for substituents of the uracil ring; also in blue, we marked the 63 obtained vertices [19]; down arrows and triangles denote those beneficial vertices (attributed to the receptor cavity), up arrows and triangles indicate the detrimental vertices, for the mono-linear and bi-linear MTD analysis of Equations (17) and (19) and the optimized charts (18) and (20), respectively; see the text for further details. Circular colors generally indicate self-corresponding visualization for the molecular group belonging in the hypermolecule.

The 1-[(2-hydroxyethoxy)-methyl]-6-(phenylthio)thymine congeners (HEPT ligands) [73,74] (see Figure 2) have proven in vitro antiviral activity, particularly against HIV-1.

An exclusive anti-HIV-1 activity of HEPT ligands is proven, at the level of viral replication. Unfortunately, there is no proof of the inhibition of other retroviruses, including HIV-2, using HEPT ligands. Research [75] concerning aspects of the synthesis of HEPT derivatives has shown that if there is a methyl group in position C-5 (*R*_1_ region in Figure 2), a cyclic structure in position C-6 (*R*_2_ region in Figure 2), it is necessary to have also a two-atom HN-3 constellation to express a powerful anti-HIV activity. 

In their work, Tanaka et al. [76] showed that derivatives HEPT-S express an increased potential of antiviral activity. Inhibition of viral *reverse-transcriptase* (*RT*) represents an attractive target to assess the anti-HIV activity of specific ligands; thus, it was challenging to point out anti-HIV activity in the case of assessing the impact of different substituents on the phenyl ring and upon C-5 [76]. In Figure 2, on the general structure of HEPT are represented the effects of the presence of certain atomic groups at the level of certain positions within HEPT.

The minimal topological difference (MTD) method translates data into 3D. It offers several advantages in terms of molecular modeling techniques, starting from a correlation equation of higher/improved quality, having as a result the obtaining of those specific stereochemical conditions in order to optimize the anti-HIV-1 attack [77].

Substituents in the C-5/*R*_1_ and C-6/*R*_2_ positions on the phenyl ring via oxygen bounds or via the sulfur bridge at the level of C-6/*R*_2_ are solid reasons that determine the investigation of the anti-retroviral activity of HEPT series [73,74,78,79]. This means that the mentioned positions of substituents with various possibilities for them, but not 1-[(2-hydroxyethoxy)-methyl], provide the inhibition of the *reverse-transcriptase* (*RT*).

In Figure 2, we represent the general structure of a series of HEPT derivatives consisting of 79 congeners. We used the HyperChem 7.01 program package [80] to obtain the most stable conformations, which means the congeners with the lowest energy. Superposition was performed on the pyrimidine-2,4(1*H*,3*H*)-dione cyclic structure, in order to obtain the minimal distance between equivalent atoms. If this distance exceeds 0.5 Å, a new vertex has been introduced in the hyper-molecule. This process led us to obtain a hyper-molecule containing 63 vertices.

We obtained the *S*^0^ beginning chart in an automatic fashion. The principle that provided this assumed that there is a relation between the degree of the molecular activity at the local level and the occupancy of vertices taken individually. Therefore, the *S*^0^ beginning chart is represented as follows [19]:

(10) $$S^{0}=\left\{ \begin{array}{l} {\text{(ε}}_{j}=-\text{1): 28,35,40,42,53,54,55,56,57,60,61,62,63}\\ {\text{(ε}}_{j}=\text{0): 1,2,3,4,5,6,7,8,9,10,15,16,17,18,19,20,21,22,}\\ \;\;\;\;\;\;\;\;\;\;\;\;\text{27,32,33,34,37,38,41,45,46,49,50,51,58,59}\\ (\varepsilon_{j}=+\text{1): 11,12,13,14,23,24,25,26,29,30,31,36,39,43,44,47,48,52} \end{array}\right.$$

The obtained QSAR model, as for each QSAR model, has to be validated; we used the *cross-validation* (*cv*) technique. It involves several steps: first, a series of ligands, *N*, has to be considered with the calculated activities. This series is split into two sub-series, odd and even. The odd sub-series determines the S* optimized chart, as well as the correlation equation. The even sub-series determines the MTD_i_ parameters and the linked *A*_i_* activities. Reciprocity is the principle that concludes this model: activities of the molecules in the odd sub-series are derived from those of the even sub-series. The cross-validation correlation index:

(11) $$r_{\text{cv}}^{2}=1-\frac{\sum_{i=1}^{N}{\left(A_{i}-A_{i}^{*}\right)}^{2}}{\sum_{i=1}^{N}{\left(A_{i}-\bar{A}\right)}^{2}}$$

is obtained through its $100\cdot r_{\text{cv}}^{2}$ value. Equation (9) and S* contribute to predicting a proportion of the variance of the experimental biological activity, with an $\bar{A}$ average.

QSAR models of considered HEPT congeners are analyzed as follows, by means of their experimental anti-HIV activity [73,74,78,79]:

- the mono-parametric model of hydrophobicity:
  (12) $$A_{i}=\alpha_{0}+\alpha_{1}\log P_{i}$$
- the mono-parametric model when the steric parameter is:
  (13) $$A_{i}=\alpha_{0}-\beta \cdot {\text{MTD}}_{i}$$
- the combined correlation:
  (14) $$A_{i}=\alpha_{0}+\alpha_{1}\log P_{i}-\beta \cdot {\text{MTD}}_{i}$$

We used the HyperChem program package [80] and the option “QSAR Properties” in order to compute the parameters that define hydrophobicity in Equations (12) and (14). MTD_i_ parameters are those that are in relation to the S* optimized chart of the receptor; regression parameters α_0_, α_1_ and β describe the obtained QSAR studies. *A*_i_ is the calculated value of *A*_exp,i_.

The algorithm of hyper-molecule optimization starts from the *S*^0^ beginning chart, *S*^0^ = {ε_j_}_j=1,..., M_. Attribution of vertices (ε_j_) within the hyper-molecule is done in a consecutive manner: initially, the *r_0_* correlation coefficient is taken into account (it is linked to *S*^0^); the process is repeated until the maximal correlation coefficient *r*_max_ is obtained that describes the S* receptor’s optimized chart. It is worth mentioning that every *r* is the result of the minimization process of the sum of squares of the differences between experimental and calculated biological activities:

(15) $$\text{Y=}\sum_{\text{i=1}}^{N}(A_{i}-A_{\text{i,calc}}{)}^{2}$$

We can state that we have obtained an optimized receptor chart S* in the situation when any new change of ε_j_ does not produce any change (decrease) of (15). In a simplified manner, S* is the picture of the interaction between *R* and *L*_i_ ligands.

Hydrophobicity has the following regression equation type [19]:

(16) $$A_{i}=3.838(\pm 0.327)+0.790(\pm 0.099)\cdot \log P_{i}\;(N=79;r=0.673;F=63.85)$$

Its interpretation is logical and simple: biological activity increases with the increase of the partition octanol/water. Although the correlation coefficient records a good value, its impact is rather frugal, meaning that in this kind of QSAR models, it is necessary to consider also steric effects: hydrophobicity should be excluded, and MTD parameters should be considered or MTD parameters should be part of Equation (16).

In the case of the 79 HEPT biologically-active congeners, the mono-linear MTD Equation (13) provided an improved statistical model that registered an improved correlation coefficient (*r* = 0.830) [19]:

(17) $$A_{i}=15\text{.894(}\pm 0\text{.742)}-0\text{.834(}\pm 0\text{.064)}\cdot {\text{MTD}}_{i}\;(N=79;r=0.830;F=169.9;r_{CV}^{2}=0.685)$$

Figure 2 shows the hyper-molecule’s optimized chart and the vertices’ attribution, S*; it is nevertheless unfolded from Equation (17) through employing the minimization procedure described by Equation (15) and looking like [19]:

(18) $$S_{\text{MTD}}^{*}=\left\{ \begin{array}{l} {\text{(ε}}_{j}=-\text{1):21,24,28,40,42,53,54,55,56,60,61,63 }\\ {\text{(ε}}_{j}=\text{0): 1,2,3,4,5,6,7,8,9,10,12,14,15,16,17,18,19,20,}\\ \;\;\;\;\;\;\;\;\;\;\;\;\text{25,27,32,34,35,36,38,39,45,49,50,51,58,59}\\ {\text{(ε}}_{j}=+\text{1): 11,13,22,23,26,29,30,31,33,37,41,43,44,46,47,48,52,57,62} \end{array}\right.$$

Now, the chart (18) gives knowledge about the stability of the vertices’ attribution in the **H** hyper-molecule while quantifying the place of ligand-receptor binding as: inside the cavity… (−1), inside the walls… (+1) and corresponding to the steric irrelevant region… (0).

The results are obtained using the mono-parametric MTD method (17); they nevertheless prove that the substituents’ presence in the *R*_1_ region (C-5 of Figure 2) explains the interaction only when they are of relatively low dimension (i.e., methyl, ethyl, isopropyl, cyclopropyl). Note that the more voluminous substituents (e.g., allyl, propyl, phenethyl and benzyl) induce the appearance of detrimental vertices, with negative influence on the anti-HIV activity.

On the other side, in the *R*_2_ substituents’ region (C-6 of Figure 2), the phenyl rests interact positively with the receptor site once they are substituted in *meta* positions with small radicals; the detrimental effect is carried here by the methoxy, methoxycarbonyl, nitro and cyano radicals.

Overall, the *R*_3_ region (N-1 of Figure 2) is the most favorable region for receptor interaction. Accordingly, the benefic effect on the anti-HIV activity is recorded by single and voluminous substituents bound to the 2-hydroxyethyl rest; these substituents are mostly aromatic, so interacting with a relatively large receptor cavity by means of van der Waals (hydrophobic) bonds [81]. The mentioned hydroxyethyl rests in “extreme” regions develop detrimental vertices in the region.

Finally, one considers both the hydrophobicity parameter (Log*P*) and the minimal topological difference towards the formation of a bilinear statistically-validated Equation [19]:

(19) $$A_{i}=13\text{.844(}\pm 0\text{.972)}+0\text{.488(}\pm 0\text{.070)}\cdot \log P_{i}-0\text{.674(}\pm 0\text{.064)}\cdot {\text{MTD}}_{i}\;(N=79;r=0.882;F=133.2;r_{CV}^{2}=0.774)$$

note that the correlation coefficient, *r* = 0.882, is significantly improved respecting the simple case of the mono-hydrophobic model (16). Therefore, it is computationally proven (and presumed valid not only in this exposed case) that the fundamental feature of the MTD-QSAR contribution is in enhancing the degree of correlation as based on the accounting of the steric effects.

Moreover, Equation (19) tells us that while high values of the MTD parameters relate to the structural and bonding symmetries’ effects in decreasing the biological activity; the opposite situation occurs when the complementary symmetry is parameterized. Accordingly, higher biological activity correlates with the asymmetry degree of the hyper-molecule, a feature appropriately described by the MTD steric quantifications

Actually, by using Equation (19), the optimized MTD chart of parameters can be obtained as before as [19]:

(20) $$S_{\text{MTD}+\text{logP}}^{*}=\left\{ \begin{array}{l} {\text{(ε}}_{j}=-\text{1):14,21,28,36,39,40,42,49,53,54,55,60,61,63 }\\ {\text{(ε}}_{j}=\text{0): 1,2,3,4,5,6,7,8,9,10,12,15,16,17,18,19,20,}\\ \;\;\;\;\;\;\;\;\;\;\;\;\text{26,27,32,33,34,35,37,38,45,48,51,58}\\ {\text{(ε}}_{j}=+\text{1): 11,13,22,23,24,25,29,30,31,41,43,44,46,}\\ \;\;\;\;\;\;\;\;\;\;\;\;\;\;\text{47,50,52,56,57,59,62} \end{array}\right.$$

The interpretation of Equation (20) is made in relation to Figure 2: the vertices’ attribution in the hyper-molecule has been changed respecting the inset case, especially in the *R*_3_ substituents’ region. In fact, the vertices 14 and 49 as being part of the “extreme” vertices of the hydroxyethyl rest became benefic, while the vertices 56 and 59, corresponding to voluminous rests as phenyl and cyclohexyl, became detrimental. It is also possible that the hydrophobic interactions (appearing when in the same region, other voluminous rests are considered) do not exist, whereas hydrophobic interaction favoring van der Walls interactions in the mentioned positions (i.e., the vertices 53–55, 60 and 61). Most probably, the simultaneously increase of biological activity along the octanol/water partition coefficient is due to an accelerated transport of ligands to the receptor site; a matter topologically sustained by the MTD parameter characterizing the ligand-receptor interaction.

The last idea is crucial for the future of special QSAR and special computational chemistry: including the hydrophobicity and topological influence by MTD parameterization significantly improves the chemical structure-biological activity correlation and the allied equations’ qualities; yet, it does not sensibly modify the hyper-molecule vertices’ effective contribution. However, the peak change is shown in the *R_3_* substituents’ region, where the voluminous rests close to the *R_2_* region contain vertices with detrimental interaction. Overall, the multi-parametric models’ analysis may be considerably improved when, apart from combining the structural descriptors of different classes, one adds also the topological contribution, including the hydrophobicity parameter to the correlation coefficients close to 0.9 [19].

## 4. New Pharmacophores in Severe Genetic Disorders

### 4.1. 3D-QSAR Modeling for Severe Genetic Disorders

In the last few years, advanced QSAR techniques (alignment-dependent comparative molecular field analyses, comparative molecular similarity indices, alignment-independent comparative molecular field analyses or SPECTRAL-QSAR) were widely used in the design of pharmacophores [14,20,21,82,83]. 

In CoMFA techniques, two critical molecular descriptors are used for describing the interaction between ligands and cellular targets: steric and electrostatic. In addition, CoMSIA considers hydrophobic and hydrogen donor/acceptor bond properties as being critical for drugs’ biological activity. Supplementary to this, 3D-QSAR-ALMOND considers that many types of molecular descriptors are important for drugs’ interactions, for instance the interactions of drugs with different species of ions (sodium, potassium, calcium, iron, etc.). Multivariate statistics methods (e.g., partial least squares (PLS)) are applied [10]. Statistical parameters that validate the QSAR model are represented by SD (standard deviation), *q*^2^ (cross-validated *r*^2^), *r*^2^ (correlation factor), SEE (standard error of estimate) and *F* (Fischer) [10,11]. Avram et al. [20] presented a recent review of the molecular descriptors that appeared to be critical in the interactions of antidepressants with membrane receptors. We summarize a few molecular descriptors of antidepressants in the interaction with membrane receptors in Table 1.

Equally, QSAR studies applied to the interactions of neuroleptics and membrane receptors identified the same critical molecular descriptors mentioned above. In Table 2, we present a few of them in a selective manner.

Recently, an extension of classical QSAR methods applied to small molecules was used to predict the interactions among macromolecules, like proteins [89]. It was noticed that the important molecular descriptors associated with significant protein–protein interactions were:

- the van der Waals term of the potential energy;
- van der Waals surface area and, also, solvent accessible surface areas calculated as an approximate sum of all hydrophobic atoms’ contributions [15,90];
- counts of bonds from the structure of interest [91];
- dipole moments, both electronic and hydrophobic [92,93,94]; and
- van der Waals surface areas of polar atoms and, also, hydrogen bond acceptor atoms [90].

The hydrophobic moment of proteins is a critical descriptor, especially when changes in proteins’ conformation are of interest [92,93,94]. When investigating the hydrophobicity of proteins with known 3D structures, a strong periodicity can be identified in most cases of amino acid sequences folding as α-helices (3.6 residues) or as strands of β-sheets (2.3 residues). The hydrophobic moment is a sum over the product of the hydrophobicity of each amino acid and the distance in space between its own and the whole protein centroid.

The binding of proteins to small ligands and even to other proteins requires interactions that involve a molecular surface. In molecular modeling, this surface can be described as the van der Waals surface area and solvent accessible surface area, and the parameters chosen for calculating them can have a great impact on the estimation of various protein properties important for understanding protein-protein interactions or proteins folding [90]. When simulating proteins, surface area calculation is difficult due to the large number of polar and hydrophobic atoms from the protein composition, each of them having different acceptors/donors of hydrogen bond descriptors. Such problems were already discussed in the literature [90]. 

The molecular descriptors presented in this section clearly suggest that, in the body, molecular structures of drugs impose their functions, and any changes of their chemical structure may induce changes in their functions. Based on our expertise in computational chemistry and in the design of new pharmacophores with wide applications in psychiatric disorders’ treatments, in the next section, we will discuss a few recent studies focused on changes in the chemical structure of drugs able to induce brain disorders and important rules in the generation of new potent pharmacophores.

### 4.2. 3D-QSAR Predictions in Pharmacophores’ 3D-Interaction

Psychiatric disorders like depression and psychosis are severe mental disorders induced by disturbance in the neurotransmitters’ balance in the brain and also by genetic risk factors. The clinical studies conducted during the last few years mentioned an increase in the cases of patients that suffer from psychotic depression [95]. Depression symptoms are low mood, behavior perturbations and alterations of the cognitive processes and suicidal behaviors [96,97]. Besides these, some disturbances of normal psychological and functional features appear (e.g., somatizations) [96]. Schizophrenia symptoms are generally classified into two classes: positive (e.g., hostility, hallucinations, grandiosity and life disorganization) and negative symptoms (decreases of attention, affective disorder, several degrees of social impairment) [98]. Recently, it was shown that many patients diagnosed with psychotic depression depend on strong treatments, based on antidepressants and antipsychotic drugs, simultaneously. The most prescribed treatment in psychotic depression are antidepressants that belong to the serotonin-reuptake inhibitor class (sertraline, paroxetine, fluvoxamine, escitalopram) [99] and other drugs used for schizophrenia treatment (e.g., typically, like chlorpromazine haloperidol, and atypical, like olanzapine, sertindole, risperidone and quetiapine) [100]. In the last few years, many other chemical structures expressing antipsychotic activities were reported [12,21,101,102]. The action mechanisms of drugs involved in psychotic depression therapy consist of the blockage of several types of membrane receptors, with a major impact on dopamine (D2, D3, D4), serotonin receptors (5-HT1A, 5-HT2A) or on the serotonin transporter (SERT) [13,101].

The most prescribed antidepressants and neuroleptics in psychotic depression are presented in Table 3 in a selective manner.

The genetic risk factors of psychiatric disorders represented by specific molecular mechanisms of genetic errors are yet unclear. It is know that identical copies of chromosomes are obtained during mitotic cell division. Correct sister chromatids pairs are produced if the bipolarity of chromatids and centrosome duplication are achieved [103,104]. During these processes, the anaphase is delayed by a complex of proteins, namely the mitotic spindle assemble checkpoint (SAC), until all pairs formed by sister chromatids go through the biorientation process [105,106]. SAC proteins, like mitotic arrest-deficient proteins (Mad1, Mad2) or serine/threonine protein kinase, are highly conserved throughout species evolution [107,108].

They present a complicated interaction pattern. In a synthetic description, Mad2, a protein that natively has two conformers, open-Mad2, corresponding to an inactive state, and closed-Mad2, corresponding to an active state, is activated by the Mad1-Mad2 core complex [109,110]. Afterward, in complex with Mad1, closed-Mad2 recruits cytosolic open-Mad2, which further interacts with Cdc20. The Mad2-Cdc20 complex binds BubR1-Bub3 to Cdc20, forming the APC/C-inhibitory mitotic checkpoint complex [111,112]. Yang et al. [110] showed that Mad2 mutants are able to adopt the two different spatial conformations and to interact in different manners with Cdc20. 

Considering that the number of computational studies on the QSAR-Mad2 protein is low, Avram et al. [89] calculated the molecular descriptors of critical Mad2 mutants. Their study represents an original approach of applying QSAR methods to large molecules like Mad2 native and Mad2 expressing mutations with the purpose of linking Mad2 features with their biological role played during chromosome segregation. In this study, QSAR methods were successfully applied to establish a correlation between the predicted properties of the Mad2 protein family and the following experimentally-determined features: (i) binding to Cdc20; and (ii) the interconversion rate from nAMEO-Mad2 to C-Mad2 configurations. Three QSAR models were derived, and their biological prediction power is shown by appropriate statistical parameter values; see Table 4.

Some important conclusions regarding the molecular descriptors of the Mad2 protein critical for its functions were: (i) the most relevant descriptors that are useful to predict the role of Mad2 and mutant chromosomes’ instability are: van der Waals area, water accessible surface area and their subdivided, van der Waals energy; (ii) protein hydrophobic and dipole moments, as well as van der Waals surface areas over polar and hydrogen bond acceptor atoms are critical in computing the prediction of Mad2 mutants as inductors of chromosome instability.

It was suggested that the molecular descriptors of native and mutant Mad2 evaluated in this paper represent an important study for future studies focused on aneuploidy, provided that kinetic data about Mad1-Mad2 and/or Mad2-Cdc20 are available.

Taking into account the complexity of psychotic depression symptoms, Avram et al. [21] obtained powerful 3D-QSAR-ALMOND models able to predict simultaneously the antidepressant and antipsychotic activity against the 5-HT1A receptor. The novelty of this study was represented by the presence in the proposed QSAR models of crucial ions (potassium, calcium and iron), which potentiated the drugs’ biological activity. The presence of these ions was indicated by experimental evidence. Robust QSAR models (expressed by values of statistic *q*^2^ and *r*^2^ values) were based on combinations of descriptors: QSAR Model 1, water and sodium (*q*^2^ = 0.60, *r*^2^ = 0.97); QSAR Model 2, water and potassium (*q*^2^ = 0.64, *r*^2^ = 0.97); QSAR Model 3, water and calcium (*q*^2^ = 0.63, *r*^2^ = 0.96); and QSAR Model 4, water and iron (*q*^2^ = 0.76, *r*^2^ = 0.98). Good correlations between experimental and predicted biological activities were obtained for each QSAR model (residual values (0.01–1.77)) for all molecules.

Another recent paper [88] presented the design of a biphenyl moiety linked with aryl piperazine and syntheses of fourteen 1-(biphenyl-4-yl)-2-[4-(substituted phenyl)-piperazin-1-yl]ethanone derivatives along with their pharmacological evaluation for antipsychotic activity and computational studies, including QSAR and descriptor-based similarity study. In this study, the pharmacophoric features of biphenyl and aryl piperazine using the acetyl linkage were used to predict the atypical antipsychotic potential of the new compounds. The results of the study showed that: (i) all derivatives possess considerable anti-dopaminergic and anti-serotonergic activity, and two very important compounds, namely 1-(biphenyl-4-yl)-2-[4-(2-methoxyphenyl)-piperazin-1-yl]ethanone and 1-(biphenyl-4-yl)-2-[4-(2,3-dichlorophenyl)-piperazin-1-yl]ethanone, presented an antipsychotic profile with lower potency for catalepsy induction; (ii) the developed QSAR models showed that the predicted brain/blood partition coefficient (QPlogBB) and electron affinity (EA) correlated with antidopaminergic activity.

## 5. Conclusions

The goal of molecular modeling is beyond explaining physical and chemical phenomenon and/or properties, yet targets also new experiments’ design (meaning testing new molecular structures with pre-definite architecture). The molecular (read structural) modeling alone is not able to give a quantitative prediction of biological activity, unless it is enriched with quantum topological analysis: quantum for interaction, topological for vicinity and stericity; structural chemistry may however stand as a data-driven space guiding (by chemical intuition) the design of new molecular structures, potentially not foreseen by custom macroscopic chemical knowledge, with increased bioactive response and action [81]. 

Further 3D-QSAR studies are envisaged targeting the possibility to know the structural requirements for a compound to have an increased anti-HIV activity [19].

On the other side, recently, research on the molecular aspects of genetic disorders has been a hot topic in medicinal chemistry. Therefore, a large number of studies is focused on chromosomal instability and its possible treatments. Risk factors for chromosomal instability and a target of many genetic disorders’ understanding is the overexpression of spindle assembly checkpoint proteins. Among these proteins, mitotic arrest-deficient proteins, Mad1 and Mad2, are crucial. As a novelty, here, we present a structure-activity relationship (QSAR) study applied to SAC proteins as possible targets for the development of new pharmacogenomic studies. In the present paper, we also detail recent research in the field of QSAR applied to small chemical compounds with clinical application as last generation drugs used for genetic disorders’ treatment. The efficiency of antidepressants and antipsychotics drugs in controlling mental symptoms and also their side effects are critical subjects for the development of new psychiatric treatments. Researchers are focused on reducing side effects and understanding their pharmacodynamic/pharmacokinetic features in order to develop new pharmacophores.

## Figures and Tables

**Table 1:** Symbols and chemical and physical considerations of significant molecular descriptors of the antidepressants.

Name of Descriptors	Chemical and Physical Considerations of Descriptors	Reference
Dipole-mag	electronic descriptor involved in ligand-receptor interactions	[84]
SASA	= total solvent accessible surface area	[85]
FOSA	= hydrophobic component of the total solvent accessible surface area (saturated carbon and attached hydrogen)	[85]
FISA	= hydrophilic component of the total solvent accessible surface area (SASA on N, O, H on heteroatoms, and carbonyl C)	[85]
Glob	globularity descriptor	[85]
CoMFA, electrostatic descriptor	the electrostatic interactions between a probe atom, usually an sp^3^-carbon atom with a +1 charge, and the ligands are calculated at uniform grid points following the Coulombic function	[86,87]
CoMFA, steric descriptor	the steric interactions between a probe atom, usually an sp^3^-carbon atom with a +1 charge, and the ligands are calculated at uniform grid points following the Lennard–Jones function	[86,87]

**Table 2:** Significant descriptors and chemical and physical considerations of them when antidepressants activity is considered.

Descriptors	Chemical and Physical Considerations of Descriptors	Reference
Steric and hydrogen bonding interaction energies	the energies calculated with the water probe contain the steric and hydrogen bonding interaction energies, supplied by the presence of sodium, potassium, calcium and iron	[21]
EA	electron affinity	[88]
BBB	blood brain barrier	[88]
QPlogBB	brain/blood partition coefficient	[88]
CoMFA/CoMSIA, electrostatic descriptor	The electrostatic interactions between a probe atom, usually a sp^3^-carbon atom with a +1 charge, and the ligands are calculated at uniform grid points following the Coulombic function	[14]
CoMFA/CoMSIA, steric descriptor	The steric interactions between a probe atom, usually an sp^3^-carbon atom with a +1 charge, and the ligands are calculated at uniform grid points following the Lennard–Jones function	[14]

**Table 3:** Antidepressants and antipsychotics classifications.

Classes of Antidepressants	Chemical Structure	Molecules Name	Chemical Classes
Selective serotonin reuptake inhibitors (SSRIs)	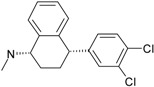	sertraline	tetrahydronaphthalenes
	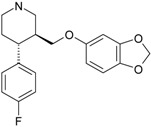	paroxetine	piperidines
	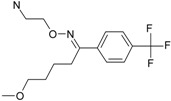	fluvoxamine	benzenes
	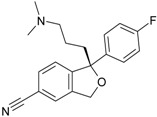	escitalopram	benzofurans
Serotonin norepinephrine reuptake inhibitors (SNRIs)	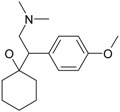	venlafaxine	phenols
	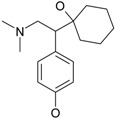	desvenlafaxine	phenols
	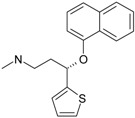	duloxetine	naphthalenes
Newer generation of drugs	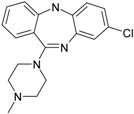	clozapine	benzodiazepines
	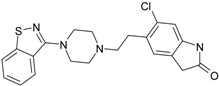	ziprasidone	phenethylamines
	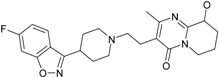	paliperidone	benzoxazoles
	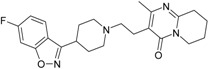	risperidone	benzoxazoles
	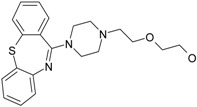	quetiapine	benzothiazepines
	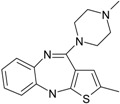	olanzapine	benzodiazepine

**Table 4:** Statistical parameters determined for the three QSAR models [89].

QSAR Models	*q*^2^ (Cross-Validated *r*^2^)	*r*^2^ (Fitted Correlation)	Root Mean Square Error (RMSE)	Cross-Validated RMSE	Fischer Test
QSAR Model 1	0.53	0.82	0.15	0.27	13.22
QSAR Model 2	0.65	0.83	0.14	0.20	10.03
QSAR Model 3	0.60	0.90	0.10	0.25	10.23

## Abbreviations

(Q)SA(/P)R	quantitative structure-activity (/property) relationships
HIV	human immunodeficiency virus
HAART	highly-active antiretroviral therapy
AIDS	acquired immune deficiency syndrome
RT	reverse transcriptase
FDA	Federal Drug Agency
CB	chemical-biological
A	activity
L	ligand
R	receptor
LR	Ligand-eceptor complex
